# Is the Kent–Rosanoff Word Association Task Helpful in Characterizing Language Difficulties in 6- to 9-Year-Old Children with Autism?

**DOI:** 10.1177/23969415261416776

**Published:** 2026-01-20

**Authors:** Carmela Miniscalco, Jakob Åsberg Johnels, Emilia Carlsson

**Affiliations:** 1 Gillberg Neuropsychiatry Centre, Institute of Neuroscience and Physiology, Sahlgrenska Academy, University of Gothenburg, Gothenburg, Sweden; 2 Department of Pediatric Speech and Language Pathology, Queen Silvia Children's Hospital, Sahlgrenska University Hospital, Gothenburg, Sweden; 3 Child and Adolescent Neuropsychiatry Unit, Queen Silvia Children's Hospital, Sahlgrenska University Hospital, Gothenburg, Sweden; 4 Institute of Neuroscience and Physiology, Speech and Language Pathology Unit, Sahlgrenska Academy, University of Gothenburg, Gothenburg, Sweden; 5 Department of Education and Special Education, University of Gothenburg, Gothenburg, Sweden

**Keywords:** Autism spectrum disorder, Kent–Rosanoff task paradigmatic, word association, autism language disorder, autism language normal

## Abstract

**Background and Aims:**

Previous research has suggested that a word association task called the Kent–Rosanoff task, which measures syntagmatic and paradigmatic associations, can help detect language disorder (LD) in children. We aimed to investigate the performance pattern between the results of the Kent–Rosanoff word association task and other language measures in two groups of children with autism: (1) children with autism and language disorder (ALD) and (2) children with autism and average language ability (ALN).

**Methods:**

Forty-six children aged 6 to 9 years (9 girls, 37 boys) with autism participated in a comprehensive language assessment that focused on receptive vocabulary, receptive grammar and sentence repetition, and nonverbal cognitive ability. The children with autism were divided into two subgroups, namely ALD and ALN, based on language ability while the groups did not differ in nonverbal cognitive ability. The 50-item Kent–Rosanoff list was used to elicit word associations. The children's responses were categorized into four different categories: phonological, syntagmatic, paradigmatic, and other/no response.

**Results:**

Our results did not reveal any differences between children with ALN and ALD on the Kent–Rosanoff word association task. The most frequent category coded for both groups was paradigmatic responses.

**Conclusions:**

Children with ALD did not differ in their semantic associations when compared with the ALN group; thus, sources of diversity in language profiles in preschoolers with autism should perhaps be sought elsewhere or by using alternative measures.

**Implications:**

Our results show that the Kent–Rosanoff word association task did not distinguish between ALN and ALD. Future studies should continue exploring sensitive assessment formats of semantic depth in young children with or without LD and autism.

## Introduction

Currently, there is no widely accepted model of how language difficulties may interact with autism characteristics and cognitive abilities (Schaeffer et al., 2023) although the heterogeneity in language ability in children with autism is well-known (Boucher, 2012; Gernsbacher et al., 2016; Schaeffer et al., 2023). All children with autism have difficulties with social interaction/pragmatics, by definition, but the condition can present with either normal language (ALN) or with language disorder (ALD) which is also specified in current diagnostic manuals (APA, 2013; Kjellmer et al., 2018; Miniscalco et al., 2024; Schaeffer et al., 2023). Schaeffer et al. (2023) presented a multidimensional model of language that consists of several distinct domains and subdomains: (1) lexicon/vocabulary (storage of words and their properties, (2) structural language (phonology, morphology, syntax, and compositional semantics [derivation of meaning from the structure of words, sentences, and larger units]), and (3) pragmatics (use of language in linguistic and nonlinguistic contexts). It was earlier believed that all children with autism had semantic problems, but research showed that their lexical knowledge varies greatly (e.g., Norbury, 2005; Reindal et al., 2023; Sukenik & Tuller, 2023).

Some research suggests that children with autism have different lexical development compared to typically developing children. A longitudinal study following children with autism from 6 to 36 months showed that they differed from typically developing children and had slower trajectories of vocabulary development with great variability in individual growth patterns (Hart & Curtin, 2023). Research has shown that while children with autism have smaller receptive vocabularies than children without autism, they produce a similar proportion of the words they understand (Artis & Arunachalam, 2023; Belteki et al., 2022). The study by Artis and Arunachalam (2023) found that children with autism show similar patterns of word use to those of typically developing children, despite smaller receptive vocabularies. In addition, they did not differ compared to children with typical development regarding syntactic properties or the use of nouns and non-nouns. Recently, a review study of lexical-semantic abilities in children with autism showed contradictory results. The review included a total of 32 empirical studies conducted in the last 10 years, with almost 50% of the included studies showing that children with autism have average lexical abilities. Most of these studies measured vocabulary size rather crudely, usually with tests of receptive vocabulary (Sukenik & Tuller, 2023). However, receptive and expressive vocabulary is only one aspect of lexical knowledge referred to as a measure of semantic breadth. Speech and language pathologists (SLPs) often assess semantic breadth by using validated measures of lexical comprehension or production of vocabulary size. Another aspect of lexical knowledge is semantic depth which refers to lexical quality or organization (Cox Eriksson, 2021). Lexical organization refers to how much knowledge an individual has about the word, how the lexicon is structured, based on phonological and semantic principles, and what kinds of networks the words include (Meara, 1996). Meara (1996) points out the importance of examining lexical organization and not just vocabulary size, as it is possible to have a large lexicon without this being effectively organized.

McGregor et al. (2013) followed vocabulary development in children with and without developmental language disorder (DLD) longitudinally (in grades 2, 4, 8, and 10). Children with DLD had vocabulary deficits characterized by limited breadth and depth compared to children with normally developing language. These deﬁcits persisted throughout the school years. In another study, McGregor et al. (2012) tried to disentangle the syntax—lexicon relationship in 9- to 14-year-old children with ASD, autism spectrum disorder and language impairment (ASDLI), and DLD (previously specific language impairment). These groups were then compared to one age-matched group and to one younger syntax-matched group regarding their verbal responses on two semantic depth measures (one phrase definition task of 40 stimulus words and one word association task). The association task comprised 40 high- and low-frequency nouns and verbs. The result showed that children with ASDLI performed similarly to peers with DLD on the two semantic depth measures.

The associations a child makes are assumed to give clues to the underlying organization of the lexicon. Lexical organization is a process that is presumed to occur in three stages: phonological, syntagmatic, and paradigmatic (Richards et al., 1985 cited in Namei, 2004). The word *dog* can thus be associated with rhyming words such as *dog*–*log* or alliterations like *dog*–*dig* but can also be nonsense words like *dog*–*tog*. Later, the child acquires more and more syntagmatic associations, which means that the words are semantically related to each other but from different form (word) classes such as *dog*–*barks*, or *dog*–*soft*, that is, that syntactic sequences, clauses, and phrases can be formed with the associated words, for example, “the dog barks,” “the dog is soft” (Schoonen & Verhallen, 2008).

Children develop a more efficient lexical organization with increasing age. The associations then become mostly paradigmatic, which means that the words are within the same word class and in a hierarchical relationship to each other, either by being superior [animal] or subordinate [dog] (Cronin, 2002). This so-called syntagmatic–paradigmatic shift occurs between ages 6 and 10 years (Namei, 2002) and is closely associated with literacy acquisition (Cronin, 2002). In Sweden, lexical organization is not routinely assessed by SLPs in children with language difficulties due to a lack of assessment tools. However, one tool used in research is the Kent–Rosanoff word association list, which assesses semantic depth hierarchically (Kent & Rosanoff, 1910). The original Kent–Rosanoff word association list comprised 100 words and was initially developed as a personality diagnostic tool for adults at the end of the nineteenth century. Since then, it has been used in several research studies focusing on lexical competence in mono- and bilingual children (Holmström, 2015; Namei, 2002; Schoonen & Verhallen, 2008).

In a rather recent Swedish study by Sandgren et al. (2021), 15 children with DLD and 35 children with typical language development (aged 6 to 9 years) were assessed with the Kent–Rosanoff word association task. The results showed significantly lower semantic depth scores in the DLD group compared to their typically developed peers. However, further exploration is needed to establish whether the task is useful for assessing semantic language in children with autism. Also, methodological concerns regarding studies of word association have been raised more generally, due to inconsistency regarding data collection and analyses (Fitzpatrick & Thwaites, 2020; Fitzpatrick et al., 2015), so further research probing the usefulness of such tasks is well motivated.

### Aim and Research Question

The main aim of the present study was to examine whether performance patterns on the Kent–Rosanoff word association task differ between children with autism with or without concomitant language disorder (LD).

The research question was:

1. Do children with autism and language disorder show difficulties in semantic depth when elicited using the Kent–Rosanoff list?

## Materials and Methods

### Participants

In total, 46 children, 6 to 9.4 years of age (9 girls, 37 boys) with autism diagnosis participated in the study. All children included had been followed since they were screened positive for autism at the age of 2.5 years, and they have been a part of a previous longitudinal study (Miniscalco & Carlsson, 2022). Thirty-two of the participating children have at least one parent with Swedish as their native language, and 14 (approximately 30%) have two parents with a different native language than Swedish. However, all parents of the participating children rated Swedish to be their child's best language. According to the Swedish National Agency for Education, 25% of the children in Swedish preschools have a foreign background (Skolverket (National Agency for Education), 2022). Parental educational/employment data were collected for mothers (*n* = 38) and fathers (*n* = 39), some data was missing. Among mothers, 4 (11%) were unemployed, 13 (34%) were employed in nondegree professions, 2 (5%) were students, and 19 (50%) held positions requiring a university degree. Among fathers, 6 (15%) were unemployed, 22 (56%) were employed in nondegree professions, and 11 (28%) held positions requiring a university degree.

#### The Autism Subgroup Assignment

Selection data and background measures on language and nonverbal cognitive measurements are presented in Table 1.

**Table 1.** Descriptives for Background Variables (Language and Cognitive [Means, SDs, Range]) in Two Groups: ALN (Autism and Average Language) and ALD (Autism and Language Disorder).

Selection and Background Measures	ALN *n* = 21 *M* (SD) Min–max	ALD *n* = 25 *M* (SD) Min–max	*p* Value	Effect Size (Cohen's *d*)
Receptive grammar^a^	91.5 (20.7) 55–116	65.0 (11.9) 55–96	**<**.**001**	−1.60
Receptive vocabulary^b^	108.2 (27.8) 68–172	69.5 (14.4) 33–92	**<**.**001**	−1.79
Sentence repetition^c^	10.2 (4.9) 1–19	5.4 (3.4) 1–13	**<**.**001**	−1.16
Nonverbal cognitive^d^	51.8 40–68	48.5 40–67	.80	−0.14
Chronological age^e^	7.4 (1.2) 6.0–9.8	7.7 (0.6) 6.6–8.9	.14	0.32

*Note*. Bold values indicate *p* <.05.

a Test of Reception of Grammar-2 (TROG-2) (Bishop, 2003; Swedish version, 2009): standard score (*M* = 100, SD = 15).

b Peabody Picture Vocabulary Test-III (PPVT) (Dunn & Dunn, 1997): raw score (min–max: 0–204).

c CELF-4 (Clinical evaluation of language fundamental-4), subtest Recalling Sentences (Semel et al., 2013): scaled score (*M* = 10, SD = 3).

d WASI (Wechsler abbreviated scales of intelligence), subtest Matrices/matrix reasoning (Wechsler, 1999): *t* score (*M* = 50, SD = 10).

e Raw score.

To be included in the study, a nonverbal cognitive ability score within the normal range (*T*-score >40) was needed according to Wechsler abbreviated scales of intelligence (WASI) matrices (Wechsler, 1999). The children were next divided into two subgroups, namely ALD and ALN, based on language ability, as determined by scores on Test of Reception of Grammar-2 (TROG-2; Bishop, 2003, Swedish version, 2009). A standard score below versus above 85 was used as cut-off for group assignment. Furthermore, the two groups ALD and ALN differed significantly on receptive vocabulary (Peabody Picture Vocabulary Test [PPVT]; Dunn & Dunn, 1997) and sentence repetition (Recalling Sentences from Clinical evaluation of language fundamental [CELF-4]; Semel et al., 2013), but they did not differ in nonverbal cognitive ability (WASI matrices) (see Table 1).

### Instruments

#### Word Association Task

To assess semantic depth a modified and shortened version (Johansson & Wahlstrand, 2010) of the original 100 words Kent–Rosanoff list (Kent & Rosanoff, 1910) was used. The shortened version consisted of 50 words (35 nouns and 15 adjectives) with the same proportion of nouns and adjectives as the original list.

**Figure 1.**
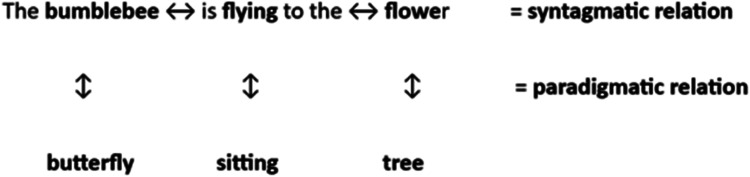
Example of syntagmatic and paradigmatic associations.

The Kent–Rosanoff list was given orally, and the single-word response was coded into one out of four categories—*syntagmatic*, *paradigmatic*, *phonological*, or *other/no answer*—with each response coded as one-point. The total score is calculated by adding up responses in the same way for all Kent–Rosanoff measures except for sematic depth. The four categories were *syntagmatic* (semantic association but not from the same lexical category, e.g., “bumblebee-flying”), *paradigmatic* (semantic association and from the same word class, e.g., “flower-tree”), *phonological*, that is, sound based (no semantic association to the stimulus word but rhyme or alliteration, e.g., bee-tree), and *other/no answer* (no semantic association to the stimulus word, e.g., “hand-owen” or no answer). Both syntagmatic and paradigmatic associations have a semantic association with the stimulus word. Syntagmatic words are exclusively of a different word class than the stimulus word, but have a syntactic connection to it. Paradigmatic responses are of the same word class as the stimulus word and have a semantic connection to it such as being in the same category, superior, or subordinate hierarchy (Figure 1).

In this study, semantic depth was calculated according to previous work by Sheng et al. (2012) and Sandgren et al. (2021). A semantic depth score was calculated by summing paradigmatic and syntagmatic associations. Paradigmatic associations were assigned 2 points, as these are considered to reflect more mature linguistic skills than syntagmatic associations, which were assigned 1 point (Sandgren et al., 2021; Sheng et al., 2012).

#### Receptive Vocabulary

PPVT-Third Edition (Dunn & Dunn, 1997) was used to assess receptive vocabulary (semantic breadth; Fyrberg et al., 2001, Swedish translation). The PPVT-III is not standardized for Swedish children, and therefore raw scores were used (min = 0, max = 204). The test consists of 204 items divided into 17 sets. Each set contains 12 items consisting of black-and-white images. Testing stops when the participant makes eight errors in one set. The raw score is calculated by subtracting correct answers, giving 1 point for correct answer and 0 point for incorrect answer.

#### Reception of Grammar

The child's receptive grammar ability was evaluated using the TROG-2 test (Bishop, 2009; Swedish version, 2013). The child had to match orally presented sentences with the correct picture out of a choice of four. The test results were presented as raw scores (number of correctly solved blocks out of a maximum of 20) and standard scores (*M* = 100, SD = 15) based on Swedish norms.

#### Sentence Repetition

The Recalling Sentences subtest from the CELF-4 (Semel et al., 2013) was used as a measure of sentence repetition. The score ranges from 0 (more than four errors) to 3 (no errors), with a maximum score of 72. The results are based on Swedish norms and presented in scaled scores around an average of 10 and a standard deviation of 3.

#### Nonverbal Cognitive Ability

The subtest Matrix reasoning of WASI was used as a measure of nonverbal cognitive ability (Wechsler, 1999). The subtest Matrix reasoning has a maximum raw score of 24 (6 to 9 years of age), giving 1 point for correct answer and 0 point for incorrect answer. Results were expressed in *T*-scores (*M* = 50, SD = 10) based on American norms, since no Swedish norms were available.

### Procedure

Two SLPs assessed all children during the follow-up visit, with one–two clinic visits lasting about 60 min each with breaks as needed, the tests included within the present study were parts of a large test battery, for more details see Miniscalco and Carlsson (2022).

The Kent–Rosanoff assessment was audio-recorded and then transcribed orthographically. In alignment with the adaptation of the Kent–Rosanoff list (Johansson & Wahlstrand, 2010), the SLP initiated the word association task with the children by stating: “When you hear a word, you come to think of other words. What is the first word that comes to mind? There is no right and wrong because everyone thinks differently. It is good if you can try to answer all the words. Do you understand? Do you want to test me? For example, if you say coffee to me, I will probably think of tea or black. Let's try it. Can you say a word and we will see what I come to think of?” Each stimulus word was then read aloud once, but if the child wished, the word was repeated once. On occasions when children were stuck on a word, they were reminded that there were no incorrect answers, and when children responded with multiple words, they were prompted to try to continue responding with only one word.

### Statistical Analysis

Group comparisons were conducted with independent samples *t*-tests and effect size measured by Cohen's *d* was calculated using the SPSS package (SPSS, IBM Corp. Released, 2021) and interpreted according to small >0.2, medium >0.5, and large >0.8 effects (Cohen, 1988). Since the sample sizes were rather small and that histogram inspections suggested that some variables deviated from normality, we also reran all analyses using nonparametric Mann–Whitney *U* tests. We report only statistical values from the *t*-tests, since all results remained the same in the nonparametric analyses.

### Ethical Considerations

The current study received ethical approval from the regional ethical review board [case number 723-13] following the 1964 Helsinki Declaration (World Medical Association, 2013). Caregivers of all children gave written consent to participation. The participants could withdraw from the study whenever they wanted without being subjected to any restrictions on further healthcare management.

## Results

Table 2 presents the outcomes obtained from the Kent–Rosanoff word association task and group comparisons between ALN and ALD. The results showed no significant differences on any measures (all *p*-values >.2). The most frequent category coded for both groups was paradigmatic responses. The less frequent category was phonological associations. Paradigmatic responses were used in 37% of the ALN answers and in 41% of the ALD group. We found that the syntagmatic responses were less frequent, 28% for the ALN group and 24% for the ALD group.

**Table 2.** Results from the Kent–Rosanoff Word Association Task (Means, SDs, Range) in Two Groups: ALN (Autism and Average Language) and ALD (Autism and Language Disorder).

Results from Kent–Rosanoff Word Association Task	ALN *n* = 21 *M* (SD) Min–max	ALD *n* = 25 *M* (SD) Min–max	*p* Value	Effect Size (Cohen's *d*)
Semantic depth^a^	50.8 (23.7) 0–94	52.7 (19.9) 2–89	.76	0.091
Associations^b^				
Paradigmatic	18.5 (11.9) 0–45	20.3 (10.1) 1–43	.58	0.17
Syntagmatic	13.8 (9.6) 0–34	12.2 (8.7) 0–30	.55	−0.18
Phonological	1.4 (1.8) 0–6	2.6 (3.7) 0–15	.21	0.38
Other/no answer	16.3 (13.3) 1–47	16.3 (12.6) 0–48	.75	−0.10

a Semantic depth was calculated by summing paradigmatic and syntagmatic associations, with paradigmatic associations assigned 2 points and syntagmatic associations assigned 1 point (Sandgren et al., 2021).

b Kent–Rosanoff list of 50 items (Johansson & Wahlstrand, 2010), raw scores.

## Discussion

The present study investigated whether the Kent–Rosanoff word association task helps distinguish between the subgroups of children with autism depending on the presence of LD. We focused on children aged 6–9 years, which is an age span commonly associated with substantial lexical reorganization and lexical knowledge during the school years (Cronin, 2002; Zarokanellou et al., 2024). Different researchers (e.g., Cronin, 2002; Namei, 2002; Sheng & McGregor, 2010) have used different word lists in word association studies. The stimulus words in these word lists have had different distributions in terms of word class and differed in terms of number of words. While not having been subject of formal studies, the types of words included in the word lists can presumably affect answers given.

In the study by Sandgren et al. (2021), which utilized the same word list as the current study, Swedish 6- to 9-year-old children with DLD scored significantly lower on semantic depth, paradigmatic, and other/no answer, but not on phonology compared to children without DLD. To our knowledge, there are no prior studies of children with autism assessed with the Kent–Rosanoff list (Kent & Rosanoff, 1910), and therefore we wanted to explore if this task could be helpful in characterizing language difficulties associated with autism. In addition, the age-range and the linguistic background (i.e., Swedish speaking) were also similar to the study by Sandgren, making the comparison meaningful. The 46 children included in our study were divided based on receptive language ability into either ALD or ALN and differed significantly on sentence repetition and receptive vocabulary (semantic breadth).

Results showed, however, that there were no significant differences between children with ALN and those with ALD in any of the four categories of the Kent–Rosanoff word association task. Thus, unlike the study by Sandgren et al. (2021), the Kent–Rosanoff test did not reliably differentiate children with autism with and without LD. The results presented in our study also did not align with those in McGregor et al. (2012) who explored and found evidence for particularly low scores in semantic depth in older children with either ALD or DLD compared to peers without LDs.

Interpreting null findings are, of course, always difficult, but it is nonetheless important to try to understand the methodological and/or theoretical groundings to the obtained pattern of results. As mentioned, the groups in our study differed in the expected direction in vocabulary performance as measured by the PPVT-III. Potentially, the results thus mirror the well described mismatch between vocabulary breadth and more complex semantic-pragmatics language abilities in autism (cf., also, Minshew et al., 1995; Zarokanellou et al., 2024). On the other hand, this would not easily explain the divergences in results compared with McGregor et al. Moreover, our findings showed rather surprisingly that both the ALD and the ALN groups answered the stimulus word more frequently with paradigmatic responses than with syntagmatic responses, thus presenting with relatively advanced skills on the task according to how it is commonly interpreted in research. The lack of a nonautistic comparison group is, however, a limitation in the interpretation of that finding.

In the study of McGregor et al. (2012) all groups with language difficulties gave fewer paradigmatic responses than the ASD group and the age matched controls. However, their five study groups included older children aged 9–14 years. In addition, the tasks differed in certain respects; for instance, although the list used by McGregor et al. contained a combination of high- and low-frequency nouns and verbs, the current version of the Kent–Rosanoff list contains nouns and adjectives that were selected for being of high frequency (Namei, 2004). All of this makes direct comparisons with our study more difficult. Irrespective of the exact reason, we argue that further studies would be needed in order to evaluate the clinical usefulness of the Kent–Rosanoff task in assessment of language and communication in children with autism.

### Strengths and Limitations

In our study, a relatively large group of children with autism was included. A limitation was that we did not have a comparison group of children with typical development, thus we cannot definitely say whether both the ALD and ALN groups performed different than nonautistic children of the same age. It is also important to acknowledge that group scores hide great individual variation.

## Conclusion

According to our results, the Kent–Rosanoff word association task may not be obviously helpful in characterizing language difficulties in 6–9-year-old children with autism. Our results show that the Kent–Rosanoff word association task did not distinguish between ALN and ALD. This result indicates that when performing this task children with ALD did not differ in their semantic associations when compared with the ALN group; thus, sources of diversity in language profiles in children with autism should perhaps be sought elsewhere or by using alternative measures, although we think future research using this task is also needed.
